# Factors for optimizing intervention programs for cognition in older adults: the value of exergames

**DOI:** 10.1038/s41514-023-00103-7

**Published:** 2023-03-29

**Authors:** Alexandra Perrot, Pauline Maillot

**Affiliations:** 1 Université Paris-Saclay, CIAMS, 91405 Orsay cedex, France; 2 Université d’Orléans, CIAMS, 45067 Orléans, France; 3 Université de Paris Cité, I3SP, F-75015 Paris, France

**Keywords:** Therapeutics, Geriatrics

## Abstract

This review presents factors that could optimize the effectiveness of an intervention program on cognitive health in older adults. Combined, multi-dimensional and interactive programs appear to be relevant. On one hand, for the characteristics to be implemented in the physical dimension of a program, multimodal interventions stimulating the aerobic pathway and muscle strengthening during the solicitation of gross motor activities, seem to be interesting. On the other hand, regarding the cognitive dimension of a program, complex and variable cognitive stimuli appear to hold the greatest promise for generating cognitive benefits and the broadest transfers to untrained tasks. The field of video games also brings interesting enrichment through the gamification of situations and the feeling of immersion. However, some gray areas remain to be clarified, notably the ideal response dose, the balance between physical and cognitive solicitation and the programs’ customization.

## Introduction

With the representation of the elderly in the general population reaching unprecedented levels, it appears necessary to find solutions that will allow older adults to retain their autonomy as long as possible. Intervention programs that generate both physical and cognitive stimulation seem to provide interesting results. We might be tempted to propose simple and easy forms of stimulation to elderly people because of their physical and cognitive deficits. However, several results observed from interventional approaches suggest that enriching, complexifying and stimulating programs preserve or even improve cognitive functions in older adults. Exergames, which are video games that generate physical stimulation, could be a relevant tool for this objective^1^. This review presents factors to optimize exergame intervention programs on the cognitive health of the elderly.

Recently, numerous studies have highlighted the relevance of interventions combining physical and cognitive stimulation. In 2013, Fissler et al. announced that such programs would induce multi-mechanism effects that could interact positively in a synergistic approach^2^. This approach is encouraged in the clinical management of elderly persons to optimize their return home^3,4^, mainly because cognitive level and brain health are determining factors in maintaining autonomy. In 2015, Erickson, Hillman and Kramer emphasized the need to understand the best way to combine cognitive training or intellectual activities with physical activity, in order to improve cognition and brain health. They then opened perspectives toward exergames^5^.

Currently, several authors report growing evidence that combined programs are superior to simple programs in older adults, with simultaneous interventions having a greater effect than sequential ones^6–8^. The systematic review by Bruderer-Hofstetter et al. find 12 effective combined programs which improved specific aspects of physical activity and/or cognitive function. Five of these combined programs are superior to physical exercises and/or cognitive training alone^6^. Results of the meta-analysis of Zhu et al., including 20 interventional studies (2667 participants), show that the combined intervention produces greater effects on overall effect size (0.22, *p* < 0.01) compared to physical exercise^8^. In the same line, Bamidis et al. argue that simultaneous physical and cognitive training should generate more benefits than simple training, especially when the program offers a socially challenging and attractive environment, like the case of exergames^9^. In 2020, Soltani et al. described exergames as a tool to combat sedentary lifestyles and deliver physical activity in a new way, through a safe, accessible and potentially home-based experience^10^. The American College of Sport Medicine even defines exergames as the future of physical activity^11^. Exergames also address some of the barriers to practice (such as travel, weather, and fatigue). Older adults enjoy practicing exergames since they facilitate long-term investment in physical practice^12^. In studies based on exergame intervention programs^13^, the feeling of safety, attraction and enjoyment of play is high, whereas the dropout rate is very low. However, much remains to be studied in order to provide the most effective and appropriate programs possible. To this end, recent literature suggests several characteristics that are worth to be considering.

### General characteristics of the exercise program

#### Multimodality

To be more effective on cognition in older adults, combined multimodal programs offer very interesting benefits. Most of the time, these programs include a combination of aerobic physical activity and cognition (for a review^14^), although some articles highlight the relevance of other dimensions as well. In his review, Barnes states that to maximize benefits, it seems relevant in the future to offer exercises that engage learning, coordination, multiple muscle groups and continuous cognitive stimulation^15^. In a very complete multimodal approach, Montero-Odasso et al. present an intervention protocol (e.g., SYNERGIC TRIAL), combining up to four dimensions: aerobic activity, muscle strengthening, cognitive training and vitamin D intake^16^. Anderson-Hanley et al. also report the importance of more compelling, motivating multimodal interventions, that address the multiple deficits associated with a lack of stimulation and physical inactivity in older adults^17^. The value of group practice is also highlighted in a meta-analysis^8^. Indeed, social engagement has previously been associated with neurogenesis and improved cognitive function^18^, as well as a more effective practice strategy with training partners^19^.

#### Simultaneity

Another important aspect is the simultaneity of programs. In 2013, Fissler et al. pointed out that several works combining cognitive training and physical activity showed no synergistic effect^2,20–22^. For example, Legault et al. examine the effect of physical activity training intervention and/or cognitive training intervention in 73 community-dwelling persons, aged 70–85 years. They find no statistically significant differences in 4-months changes in composite scores of cognitive, executive, and episodic memory functions between the different groups^21^. A possible explanation is that the two program components were not simultaneous. Fissler indicates that simultaneity is an important component of the guided plasticity facilitation framework in order to observe interaction effects. The facilitation of cognitive plasticity induced by physical stimulation appears to be greatest during or immediately after exercise, in parallel with the increase of Brain-Derived Neurotrophic Factor (BDNF) levels, which slow down 1 h after exercise^23^. Since the time window to obtain benefits from interaction effects appears to be quite small^8^, several reviews emphasize the need for simultaneity^8,24–26^.

In fact, simultaneity also places participants in dual-task situations, allowing older adults to transfer learning to new tasks^24,27–30^. Even if most of the effects of age moderators were characteristic to a specific domain (e.g., expertize in typing will not help one drive a car better), transfers to other untrained tasks were more often observed during dual-task programs^31^. Older people can be taught to flexibly allocate their resources to different stimuli, which would generate broader benefits on high-level cognitive processes than with standard programs.

#### Interactivity

Interactivity between physical and cognitive stimulation is important to generate cognitive benefits in older adults^32,33^. Interactivity produces cognitive benefits through a synergistic effect that magnifies benefits. To prevent age-related declines and to maintain brain health, continuous active interaction with the environment that engages cognitive, physical and sensory systems is necessary^34^. The relevance of this interactivity also echoes the theory of Herold et al., which encourages further development of stimuli generating connections between physical, motor and cognitive demands^35^. This theory argues for “moving while thinking” rather than “thinking while moving”. Incorporating cognitive tasks into motor acts, appears to be the most promising approach to improve cognitive reserve, rather than presenting them separately. The “embedded” cognitive task becomes a relevant prerequisite for the success of the cognitive-motor task. Moreover, this type of stimulation more closely resembles real-life situations. This proximity to real-life situations may generate greater adherence to the practice and, thus, greater benefits. Indeed, the benefits are moderated by participants’ expectations and the perceived importance of the program^2,35^. Similar to dance or tai chi^25,36,37^, exergames offer situations that naturally combine physical and interactive cognitive stimuli^35^.

To summarize, programs combining physical and cognitive stimulation seem to be relevant to generate cognitive benefits in the elderly, particularly because of their multimodality. Moreover, they can be offered simultaneously and interactively, which seems to increase their effectiveness. It is now necessary to go into more detail about the characteristics of physical and cognitive stimulation.

### Characteristics of physical stimulation

#### Physical multimodality

Although aerobic stimulation dominates much of the literature on the subject, it appears that it does not explain everything, and that multimodality is also relevant. A growing body of work argues that resistance training programs may also be effective on cognition in older adults^38–40^. Cassilhas et al. demonstrate that 24 weeks of either thrice-weekly resistance training improved memory performance and verbal concept formation among 62 community-dwelling senior men aged 65–75 years. Liu-Ambrose et al. demonstrate that an individualized home-based program of balance and strength significantly improves inhibition (Stroop test; *p* < 0.05) after 6 months among 74 seniors aged 70 years and older. Marston et al. investigate the effect of a 12-week of intense resistance training on cognitive function in 45 late middle-aged adults and observe an improvement in verbal memory (*p* = 0.02)^40^. Resistance training is known to increase the level of Human Growth Hormone (HGH) and Insulin-like Growth Factor One (IGF-1), which have a positive effect on neuronal growth and performance^31,41–43^. Therefore, it seems relevant to combine aerobic stimulation and muscle strengthening, as the HGH and IGF-1 hormones may help to enhance the effects of an aerobic program in older individuals. In addition, the recent meta-analysis by Marinus et al. argues in favor of strengthening programs or combined strengthening/aerobic programs to generate an increase in blood BDNF levels^44^. The increase in the peripheral BDNF concentrations is significant after strength training (*Z* = 2.21, *p* = 0.003) and combined aerobic/strength training (*Z* = 3.03, *p* = 0.002) but not after aerobic exercise training (*Z* = 0.82, *p* = 0.41). Single exercise is not as beneficial as a multimodal program offering aerobic, muscle strengthening, balance and flexibility exercises^15^.

Several studies defend similar conclusions, advising solicitation of the aerobic pathway and muscular resistance, while also highlighting the value of adding situations requiring complex motor skills and coordination^45,46^. Indeed, programs based on gross motor activities have shown interesting results on cognition^47–49^. Berryman et al. reveal that gross motor activities lead to equivalent improvement in executive functions, compared to combined high intensity aerobic and strength training, in a cohort of healthy older adults^47^. Gregoire et al. find that gross motor skills training leads to increased BDNF levels in healthy over 60 years old adults, significantly more than two aerobic/strength programs^48^.

Based on several works^50,51^ and on the meta-analysis of Northey et al.^52^, Torre et al.^53^ argue that such training is relevant to improve cognitive functions in the elderly and put forward the hypothesis of cognitive-motor dedifferentiation to partly explain these effects. This hypothesis states that the reduction with age of the number of specific neural networks subservient to a specific function result in stronger links between cognitive, sensory, and motor domains^54,55^. Therefore, executive functions would be solicited, during complex motor situations, more in the elderly than in younger people. Consequently, it seems worthwhile to integrate this type of solicitation in order to increase cognitive benefits for the elderly.

If we summarize the most relevant characteristics to be implemented in the physical dimension of a program, it appears that multimodal interventions stimulating the aerobic pathway and the muscle strengthening, while soliciting gross motor activities, are the most interesting ones.

### Characteristics of cognitive stimulation

#### Challenge

The literature highlights several interesting characteristics of the cognitive dimension of a program, such as the level of cognitive challenge of the task. In their meta-analysis, Gheysen et al. report that the cognitive challenge offered by the program is more important in observing significant effects on cognition than the number of sessions^25^. Physical activity combined with cognitive challenge shows significantly greater cognitive improvement (g = 0.160; 95%, CI 0.041–0.279, *p* = 0.008) and the effects are not moderated by session frequency and intervention length. Barcelos et al. point out that exergames with high cognitive demand generate greater effects than programs with low cognitive demand tasks^32^. In particular, in their Aerobic and Cognitive Exercise Study (ACES), on 64 community based older adults, they found that exergames based on an effortful cognitive challenge (i.e., exer-score) produced greater cognitive benefits than exergames with relatively passive cognitive processing (i.e., exer-tour). Repeated measures ANOVAs conducted for each of the 3 measures of executive function reveal significant effect for Color trails after a single-bout (*p* = 0.02) and for Stroop test after 3 months of exercise (*p* = 0.007).

#### Variability

Training on a variety of tasks rather than on a single type of task appears to bring global benefits and thus to improve transfers to untrained tasks^2^. However, this variability should not concern only the number of tasks proposed but also the processes solicited by the tasks and stimuli^56^. The more a training program is associated with a variety of combinations of processes involved, the greater the transfer effect observed^57–59^. For example, Perrot et al. find that the scope of benefits of an action video game is broader than those from a cognitive training game. The digit symbol substitution test, the corsi block test, the spatial relations test, and the number comparison test show significantly greater change in the action video game group, with a larger variability in required processes than in the cognitive training game with low variability^58^. Moreover, challenging and variable tasks stimulate intrinsic motivation^60^, which is conducive to long-term adherence; at the same time, it has been shown that the psychological state related to motivation facilitates brain plasticity^61,62^.

This variability can also be reflected in the way cognitive tasks are performed in intervention programs. Indeed, Kramer et al. examined the efficacy of two different multiple-task training strategies (variable priority training and fixed priority training) for the acquisition, retention, and transfer of task coordination skills for both young and old adults. They also demonstrate that individuals trained in the variable priority condition display superior transfer to untrained tasks and better retention of time-sharing skills, over a 2-mo period, than do the individuals trained in the fixed priority condition^63^. The fact that variable-priority training showed generalization of benefits to other tasks suggests that older adults may gain efficiency in a more general cognitive skill than the one being trained. The type of training would therefore generate different cognitive effects. This argument is also supported through the INTERACTIVE model^64^, which states that training modalities (repetitive or strategic) modulate training-induced neural changes. A repetitive type of training would allow a decrease in activations and thus an increase in neural efficiency, whereas a strategic type of training would generate new activations and thus a compensation mechanism^65^. The benefits of the type of training proposed depends on the level of reserve of the participants. Older people with a high brain reserve will benefit more from strategic training than those with a lower reserve, who will prefer a repetitive type of training. It appears important to take these parameters into account in order to propose the best suited programs possible.

To summarize the interesting features to implement in the cognitive dimension of a program, complex and variable cognitive stimuli seem to hold the greatest promise for generating cognitive benefits and the broadest transfers to untrained tasks. However, training modalities will have to take into account the characteristics of the participants and the objectives to be reached.

### Specific characteristics of video games

#### Gamification

The video game and virtual environment can also be a factor in additional benefits^17,33^. Video games are inherently entertaining while stimulating cognitive function^66,67^ and increasing social engagement, positive emotions, and generating synergistic effects on cognitive function and neuroplasticity. Anderson et al. investigate the impact of 3 months of cybercycling versus traditional exercise on executive function and plasma BDNF, on 63 older adults. Results reveal significant benefits for the cybercycling group on executive function (*p* = 0.002) and on plasma BDNF (*p* = 0.05), compared with the traditional exercise group^17^. Few years later, the same author with his colleagues find that the cyberbike generated more benefits on 14 community-dwelling older adults meeting screening criteria for mild cognitive impairment, than a normal bike, and that these results cannot be explained by the level of adherence (identical between the two programs)^33^. They argue that the screen generated additional benefits, through motivation and entertainment. The gameplay offered by most exergames (e.g., virtual environment, objectives, challenges, rules, biofeedback and immersion) contributes to these games’ strong appeal^68^. Adcock et al. discuss the benefits of gamification on training^34^. The game generates an enriched environment and thus additional cognitive stimulation; in fact, this environment is further enriched because it is often new to older adults, who are less accustomed to such recreation. Moreover, some studies on the effects of environmental enrichment highlight the importance of novelty for cognition and brain health^2,69,70^.

#### Variability

Another strength of video games lies in the situations proposed by most exergames, which correspond to open tasks^68^ in which the environment varies, requiring higher executive functions^71^. In these types of situations, players are required to plan, make decisions and inhibit others in order to correctly interpret the stimuli generated by their movements and their displacements in the virtual environment^72^. Therefore, the stimuli offered by video games can improve these high-level cognitive functions^68^ that are so important to autonomy in the elderly^73^. Video game input is also interesting because it can generate transfers to untrained tasks, whereas several authors have pointed out that the benefits of cognitive training were mainly seen in trained tasks and only a few in other situations^2,74–76^. For example, Ball et al. study evaluate whether 3 cognitive training interventions improve mental abilities and daily functioning in 2832 older, independent-living adults, aged 65–94 years. They demonstrate that cognitive intervention helps older adults to perform better on specific cognitive ability for which they are trained (*p* < 0.001 for all cognitive targeted ability) but no training effects on everyday functioning are detected at 2 years^76^. This could be due to greater variability in video game situations compared to cognitive training tasks^2^. Several authors also defend the hypothesis that new motor skills acquired during gameplay could generate transfers to other activities^11,77^.

#### Immersion

Another interesting characteristic to set up and explore, which is specific to video games and exergames, concerns immersion in the game. Immersion depends on the immersiveness (isolation from the real environment), inclusiveness (number of sensory modalities proposed), and realism of the proposed virtual situation^78^. The neuropsychological performance of older people increases more with virtual reality cognitive training than with a more traditional cognitive training program, suggesting that mental engagement in an immersive environment plays an important role in cognitive and especially executive improvements. Pedroli et al. evaluate the efficacy of a novel Virtual Reality-based mixed training protocol for improving executive functions and spatial memory in patients with cognitive decline. The results show a significant improvement in executive functioning in the Virtual Reality group after the training period, compared to a classic rehabilitation protocol group^79^.

Montana et al. also confirm with a systematic review on 16 studies that virtual reality-based training increases neural plasticity^80^, which improves cognitive function. Therefore, an exergame in a virtual environment will generate more cognitive improvement than an exergame with low immersion. In 2020, Huang proposes to combine an exergame and an immersive virtual environment, for a greater effect on brain health, in particular by arousing the feeling of presence, which, in turn, increases cognitive performance in inhibition and flexibility^81^. Immersive technologies, which allow players to isolate themselves from the real world and to explore a virtual environment, generate a spatial presence experience^82^. This sense of presence is when, during a video game and/or in a virtual environment situation, players feel as if they are somewhere other than where they actually physically are^83^. And it has already been shown that spatial presence in an immersive environment generates an increase in the activity of brain regions associated with cognitive functions, and particularly executive functions^84^. It seems important to increase the immersive aspect of exergames to create a strong sense of presence, which could serve as a new strategy in preventing cognitive decline.

In 2021, Temprado’s article echoed several of the characteristics highlighted so far, namely interactivity, immersion, novelty, attractiveness and challenge, and considers them to be the essential content of an exergame program to generate interesting transfers to both cognitive functions and daily activities^46^. The author also emphasizes the importance of proposing natural and spontaneous actions in an emotional context adapted to daily life (e.g., catching, hitting, intercepting moving objects, coordinating several limbs simultaneously and controlling balance).

**Fig. 1:**
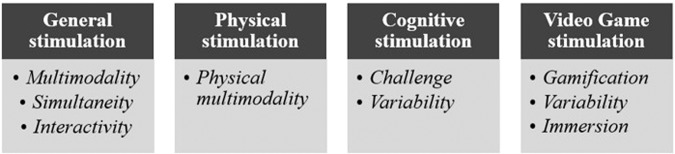
Overview of the characteristics of the stimulation programs. List of important characteristics, divided into four areas.

To summarize, Fig. 1 shows all the characteristics discussed in this review that seem to offer the most efficient stimulation to the elderly.

### Limitations and gray areas

Although several studies observe a beneficial effect of exergame programs on the cognition of older adults, that exceeds the benefits of simple programs (e.g., cognitive training, video games or physical activity) (for review^14^), the conclusions are not unanimous^85,86^ and the results remain quite heterogeneous. For example, Ordnung et al. investigate the effects of an exergame training over 6 weeks on cognitive, motor, and sensory functions in 30 healthy old participants, randomly assigned to either an exergame training group or a control group without training. Results indicate that even though exergames might improve gaming performance, behavioral assessment was probably not sensitive enough to capture exergaming-induced improvements^85^. The systematic review by Gallou-Guyot et al.^13^, conducted on 18 reviews even reports that exergames do not outperform the effects of more traditional combined programs on cognitive functions. One review finds exergame interventions effective on cognitive functions. Two reviews find that exergames interventions are equivalent compared to fall prevention programs, single task training and active and inactive controls. One reason that may explain this inconsistency is the variety of exergames that can generate different effects based on their physical and cognitive demands^87–92^. Indeed, metabolic and/or cognitive demands may be low, very basic or general in certain exergames, but highly developed and specific in others. Therefore, they can trigger different mechanisms. Three categories of exergames are identified as effectively generating differences in cognitive and physical stimulation^14^, namely (i) dance and step video games, (ii) commercial home consoles, and (iii) virtual ergometers. Each category has both positive and negative points. On the one hand, home consoles (e.g., Nintendo, Kinect) provide a wide variety of games, and thus varied forms of physical and cognitive stimulation^89^, whereas dance ergometers and exergames have more restricted variety and possible stimulation^93,94^. On the other hand, the intensity of practice is easier to control with the latter exergames, making them simpler to use in programs and to determine their effect^17,32^. Thus, there is the versatility of possible combinations of physical stimuli (endurance, strength, flexibility and motor skills) and cognitive stimuli (type of games offered and cognitive functions most solicited), which makes comparisons between studies difficult. It seems necessary to clarify the physical effort generated (intensity and type) and the cognitive demand (amplitude and functions required).

To create an optimal multimodal program for the cognitive health of the elderly, much work remains to be done to clarify not only the content but also the intensities, durations and frequencies^8,14,95–98^. Indeed, as highlighted by Erickson, Hillman & Kramer^5^, and by Gheysen et al.^25^, a considerable knowledge gap persists to understand how to get the best benefits from these enriched combined stimulations, with the dose-response relationship remaining a gray area that needs to be addressed, especially in terms of the intensities of the cognitive and physical stimulations offered simultaneously. In their meta-analysis, Zhu et al.^8^ state that stimuli that are demanding in physical intensity and cognitive demand could also have negative effects on older adults, generating cognitive fatigue^99^ excessive stress and less engagement in the activity^100^. They also find that high frequency of practice does not produce the best effects on cognition. Interventions scheduled five or more times per week showed less effectiveness than those conducted fewer than five times. These findings echo other meta-analyses conducted on cognitive training programs^101,102^ and video games^67^. Thus, it appears that a “maximum dosage” exists and that future work is needed to best adjust the frequency of practice, without risking an overdose. The beneficial effects of combined programs do not appear to depend on the length of the program, or on the number, the frequency or the duration of sessions^25,103^. More emphasis should be placed on the qualitative aspects of combined exercise and cognition programs (type of exercises), rather than on their quantitative aspects (i.e., duration and intensity)^49,53^.

At the same time, imbalances between physical and cognitive stimulation could have important consequences. It is possible that excessively high physical demand prevents older people from performing cognitive exercises and thus from benefiting from the synergistic effects. Conversely, cognitive exercises that are too demanding could limit physical investment and not reach sufficient intensity. Combourieu et al. evaluate the impact of different aerobic and cognitive training intervention programs and observed that the group that performed simultaneous aerobic and cognitive training had a significantly lower intensity of effort than the group that performed aerobic activity alone (respectively 32% and 47%, *p* < 0.05)^104^. It would therefore be relevant to explore how to achieve this right balance, related here to simultaneity, but also to compare it with very closely spaced sequential programs that could prove a suitable way to avoid the domination of one type of stimulation over another.

Several studies also emphasize the need to develop individualized exergame programs for each practitioner^11,105^.Commercially available exergames are of value because they are technically very well designed and therefore attractive and entertaining. However, they are less adaptive and specific. It is important to propose situations that are best suited to the participants’ objectives and/or abilities. The next challenges will be to propose games that are as attractive as the commercial ones, especially in terms of flow, while being fully customizable.

This part underscores that future works are necessary to clarify and to control the impact of many parameters on the effectiveness of exergames on cognitive aging. In a more general approach to the optimal program for cognition in the elderly, understanding the interactions between factors that may affect the expression of benefits is only at its beginning at present. Factors contributing to program effectiveness still require further investigation. It also seems important to further investigate how combined programs optimize the maintenance of autonomy at home and/or the return home after institutionalization. Even if the improvement of the cognitive and physical functions contributes to sustaining independence, additional work needs to be done on the transfers generated by the combined programs on activities of daily living.
